# Report of Thoracic Injuries Treated by Surgical Team No. 39 at Evacuation Hospital No. 8, from Sept. 1918 to Oct. 1918

**Published:** 1919

**Authors:** 


**Report of Thoracic Injuries Treated by Surgical Team No. 39 at Evacuation Hospital No. 8, from Sept. 1918 to Oct. 1918.** By Howard LILIENTHAL, Lieut.-Col. M. C.

There were 67 cases of thoracic injuries, or about 27 o/o of the whole number of wounded.

Conditions were as perfect as could be expected under the circumstances.

However, it is believed that the important class of abdominal and thoracic injuries might have been treated to better advantage, had there been a special operating room equipped for the purpose.

Most of the patients arrived within 24 hours after being wounded. About one-third were suffering from shock on admission. In nearly all of them a certain degree of vital depression, perhaps not amounting to actual shock, was present.

The free exploration of the thoracic cavity was reserved for those cases in which the foreign body had done such severe damage that repair was urgent, or when it was so large or so dangerously placed that its removal appeared imperative.

There were 18 cases of open sucking wounds of the thorax, with a mortality of 34 o/o, a larger death rate than in any other class of thoracic wounds. Sucking wounds, when large enough to admit air by suction, in quantities as great as or greater than that which enters the lungs through the larynx, give rise to the well-known flapping of the soft tissues of the mediastinum with each respiration. Probably the simplest method for overcoming this condition for the time-being is to close the chest in the quickest possible way.

In penetrating wounds are included cases in which the missile is within the chest, or in the thoracic wall, having passed completely through one wall of the cavity, but with no wound of exit. There was a mortality of 30 o/o.

Completely perforating wounds with entrance and exit, and no foreign body remaining, gave a mortality of 10 o/o. No large operations had to be performed in these cases. These patients usually suffer comparatively little. However, patients of this class sent to the Base will sometimes develop the most serious infection.

Bullet and shell fragments injuring the thorax were rather surprisingly similar in percentages of mortality. Bullets striking the ribs at a slant or tangentially make ugly and destructive wounds, while it is only the smaller shell fragments which give rise to injuries not immediately fatal. Small, completely perforating eclats often do little or no more damage than merely perforating machine gun bullets, while the large pieces of high explosive shells kill the men outright.

Pneumonia of the uninjured side was the commonest and probably the most serious complication. It was of the bronchopneumonic type, but the patches as discovered by physical signs and checked up by X-ray were extensive. The middle and upper parts of the lung were most often affected, the bases rarely.

In the intelligent after-treatment of chest injuries fluoroscopy in the vertical posture is a necessity. Frequently the changes in a cases had to be observed from day to day, to decide on repeated aspiration or other secondary procedures which might be of vital importance.

Bodies appearing within the heart shadow and moving with the cardiac impulse are not necessarily within the heart or pericardium. A better idea of where they will lie when the chest is open may be obtained if, after the first X-ray examination, a cannula is inserted throught the thoracic wall and left there long enough for the air to enter and the lung to collapse.

*Operative Procedures.*

*Major Intercostal Thoracotomy.* A long intercostal incision, usually in the seventh interspace, the ribs being separated from 3 to 5 inches or more by means of a powerful retractor. Resection of a rib is very rarely necessary. The lung may be collapsed or distended by means of a foot bellows through a tube in one nostril, the other nostril and mouth being closed.

*Pneumopexy.* The lung is distended by the anesthetist until it lies flush with the thoracic wall, plugging the defect. It is then neatly sutured to the edges of the pleural opening.

*Method of Emptying Hemothorax.* Patient sitting. Local anesthesia, including pleura. Incision one-half inch long through skin. Introduction of medium-sized or large cannula. Attach a rubber tube not less than 8 inches in length to the cannula after it is in place and the fluid is running. It may now be siphoned off, and, as the chest empties air, take the place of the bloody liquid. From 1000 to 2200 cc. of fluid may be quickly, safely and painlessly removed. A hemothorax emptied and shown by culture to be mildly infected may go on to recovery without operation.

Of the total of thoracic cases, 23.8 o/o died. Of those who were operated upon, 27.4 o/o died, and of those who were not operated upon, only 6.2 o/o died. This does not mean, however, that operation is responsible for the large proportion of deaths.

